# Comparing flipped classroom and conventional live demonstration for teaching orthodontic wire-bending skill

**DOI:** 10.1371/journal.pone.0254478

**Published:** 2021-07-09

**Authors:** May Nak Lau, Yasmin Kamarudin, Nor Nadia Zakaria, Saritha Sivarajan, Norhidayah @ Nor Zahidah Mohd Tahir, Aufa Dahlia Bahar, Zahra Naimie, Siti Adibah Othman, Wan N. Wan Hassan

**Affiliations:** 1 Department of Paediatric Dentistry & Orthodontics, Faculty of Dentistry, University of Malaya, Kuala Lumpur, Malaysia; 2 University Malaya Dental Education Enhancement and Development Unit (UMDEED), Dean’s Office, Faculty of Dentistry, University of Malaya, Kuala Lumpur, Malaysia; University of Macau, MACAO

## Abstract

Flipped classroom may overcome weaknesses of live demonstration in teaching orthodontic wire-bending. This study aims to compare the effectiveness between flipped classroom and live demonstration in transferring skills for fabricating Adams clasp. Forty third-year undergraduate dental students were assigned to two groups. The students in group LD (n = 20) attended a live demonstration while students in group FC (n = 20) attended a flipped classroom. Both groups were taught on skills to fabricate Adams clasp in a standardised way. Each student from both groups were asked to submit an Adams clasp for a blinded quality assessment by two trained and calibrated assessors using a 18-item rubric, followed by validated students’ satisfaction questionnaires to evaluate their perceived satisfaction on the teaching method received. A crossover study was then conducted three weeks later where LD attended a flipped classroom while FC attended a live demonstration. Students’ satisfaction questionnaires were again collected from each student for blinded analysis. Mean scores for the quality of Adams clasp were 9.775 and 9.125 for LD and FC, respectively. No significant difference was detected between the two groups. Statistically significant association was found for one statement on the questionnaire, “I found the classroom arrangements conducive for the wire-bending activity” (p = 0.010). No significant differences were found between the two groups for other statements (p > 0.05). In conclusion, within the limitations of the study, flipped classroom is equally effective as conventional live demonstration in transferring orthodontic wire-bending skills for fabrication of Adams clasp. However, students perceived the classroom arrangements during the flipped classroom significantly more conducive for teaching orthodontic wire-bending.

## Introduction

Traditionally, orthodontic wire-bending skills are taught via live demonstration in a physical classroom during the pre-clinical year in the undergraduate dental curriculum. Live demonstration is a useful teaching tool as it increases students’ confidence, enhances communication skills, and provides better understanding compared to didactic teaching [1]. However, it poses challenges for training professional skills as it is associated with some factors that decrease teaching and learning effectiveness such as difficulty in visualization of the demonstration by students, time constraints, non-repeatability, and require manpower for every demonstration to a small group of students [2, 3].

Flipped classroom is a blended learning model in which students access teaching content online prior to class, enabling interactive and collaborative activities during class to promote learning [4]. Flipped classroom provides a flexible platform for self-paced learning, thus improving students’ interest in their own learning [5–7]. We hypothesized that flipped classroom method can overcome the shortcomings of teaching orthodontic wire-bending skills via live demonstration.

While flipped classroom has been reported to be good for cognitive teaching to medical, dental, and nursing trainees [8–10], reports of the efficacy of flipped classroom for professional skills training are limited [11–13]. To date, no study has investigated the efficacy of flipped classroom on teaching orthodontic wire-bending skills. Several studies have investigated the effectiveness of video demonstration for teaching clinical and preclinical laboratory skills including orthodontic wire-bending skills [2]. Theoretically, it allows better visualisation, overcomes shortage of manpower, and enables repeated viewing before, during and after the teaching session [2]. Nonetheless, study shows that live demonstration is equally effective [2]. Systematic reviews and meta-analysis reported that flipped classroom model in undergraduate dental education was an effective way to deliver knowledge and it improves student satisfaction in majority of the studies, whilst its effect on academic scores, particularly for skill development, needs more research. In particular, time flexibility was found to be a valuable asset in this teaching and learning approach as it allowed students to assimilate the educational material at their own pace [11–13].

This study aims to compare the effectiveness between flipped classroom and conventional live demonstration in transferring skills for fabricating orthodontic Adams clasp on first permanent molar tooth to third year undergraduate dental students. The objectives were to compare the students’ satisfaction on the flipped classroom method and conventional live demonstration method on Adams clasp demonstration and to compare students’ skill in bending Adams clasp after receiving the flipped classroom method and conventional live demonstration method.

## Materials and methods

This study was a comparative prospective study conducted in the Faculty of Dentistry, University of Malaya from December 2018 to February 2019. Ethics approval was granted by the medical ethics committee, Faculty of Dentistry, University of Malaya (Ethics committee/IRB reference number: DF CD1820/0082(L)). Informed consent was obtained prior to study commencement.

**Fig 1:**
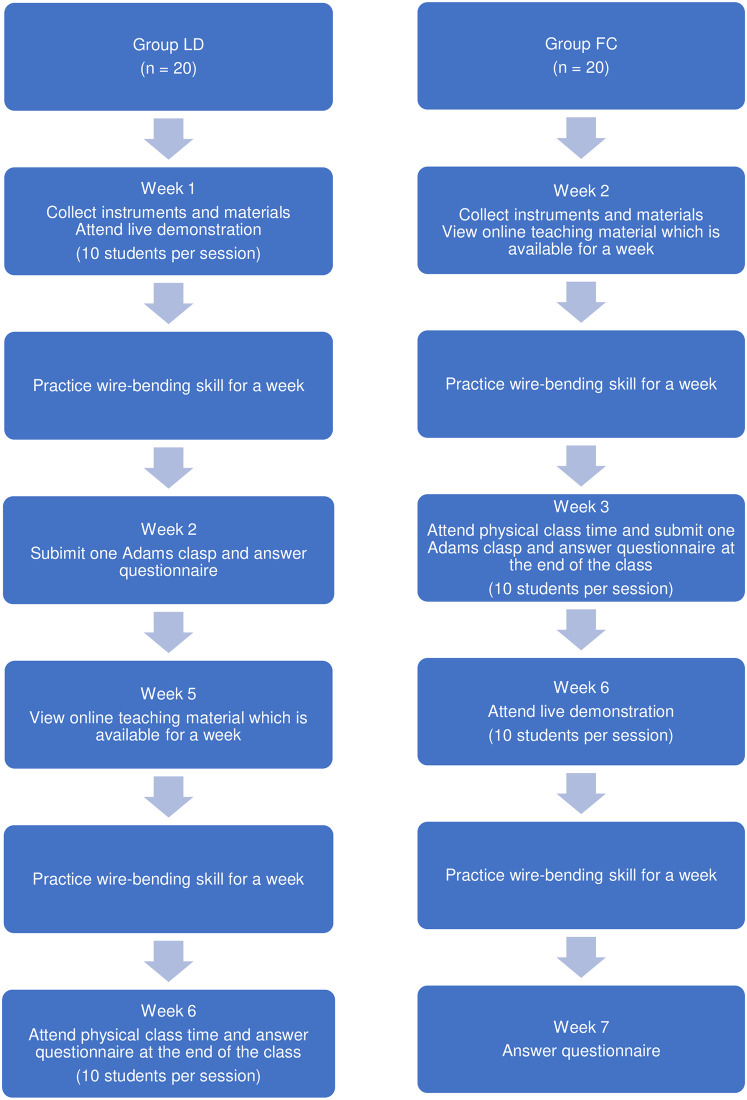
Flowchart of the research activities.

Fig 1 shows the flow chart of the study. Forty third-year undergraduate dental students, who had not been exposed to any formal orthodontic wire-bending teaching, were assigned to two groups (group LD attending live demonstration session and group FC attending flipped classroom session) based on their academic timetable. The timetable was released by the Dean’s office, Faculty of Dentistry, University of Malaya. It was randomly arranged and was not influenced by the researchers of this study. The students who were scheduled in the academic timetable to have the demonstration for Adams clasp wire-bending first, were assigned in group LD (n = 20); and the students who were scheduled in the academic timetable to have the demonstration for Adams clasp wire-bending later, were assigned in group FC (n = 20). Students in group LD attended a live demonstration while students in group FC attended a flipped classroom where online teaching material was shared to the students a week prior to a physical class time. This is to avoid students from group FC to share the teaching material posted online with students from group LD. In total, two flipped classroom sessions (10 students per session) and two live demonstration sessions (10 students per session) had been conducted for this cohort.

Both the live demonstration and flipped classroom taught skills to fabricate Adams clasp in a standardised way by the same technician providing the same instructions. For standardisation and to reduce bias, students from both groups were given one week to fabricate an Adams clasp for submission, and each group of 10 students were allocated with two instructors (one lecturer and one technician) during the classroom session. In the two-hour classroom session, students in group LD watched a live demonstration and practiced the skill with two instructors during the class time where they were given the opportunity to ask question(s). They were given the instruments and materials during the class and were allowed to take them home to continue the fabrication of the Adams clasp at their convenience. The students were required to each submit an Adams clasp a week later. Students in group FC were given access to view a video demonstration (12 minutes and 11 seconds duration) on fabrication of Adams clasp a week prior to their in-class classroom session. The video demonstration was posted on the university’s e-learning platform, the Student Powered e-Collaboration Transforming UM (SPECTRUM) where the students can watch the video demonstration using any devices at their convenience using the pause, rewind, and fast-forward functions. The university provides computers on-site but the students have their own devices which they can also access the video demonstration remotely. Students were given instruments and materials and were instructed to practice the wire-bending skill as instructed in the video demonstration throughout the week. They were allowed to watch the video demonstration and practice the wire-bending skills individually or within members of the same group. They were not allowed to share the video with the students from group LD. During this time, the students in group FC were given time to practice the skills at their own time and pace. At the end of the week, students in group FC practiced the skill with two instructors during the two-hour in-class classroom session where they were given the opportunity to ask question(s) to the instructors. The FC group were required to submit the Adams clasp at the end of the classroom session.

Both groups were instructed to fabricate the Adams clasps for a maxillary permanent first molar using identical duplicated orthodontic study model. The Adams clasps collected were then subjected to blinded quality assessment by two trained and calibrated assessors using an 18-item rubric. (see Table 1) After training, calibration was conducted by having the two assessors evaluating 10 randomly selected Adams clasps for inter-examiner reliability assessment. Both examiners examined the same 10 Adams clasps again two weeks later for intra-examiner reliability. This rubric was adapted and modified from Alqahtani et al. (2015) which originally was a 10-item rubric [2]. Details on the angle of the arrowhead, arrowhead engagement into the undercut and the direction of the tag was added in the modified rubric. Each item was graded either as poor (0 mark) or good (1 mark) adding up to a maximum score of 18.

**Table 1:** 18-item rubric for Adams clasp quality assessment.

*ASSESSMENT CRITERIA*		*Maximum possible mark*
*1*.	The bridge of the clasp should be straight	1
*2*.	The bridge of the clasp should parallel to the buccal cusp	1
*3*.	The bridge of the clasp shouldn’t touch the buccal surface of the 1st molar	1
*4*.	The height of the bridge of the clasp should be at halfway up the buccal surface of the molar	1
*5*.	The mesial arrowhead should be at 45°	1
*6*.	The distal arrowhead should be at 45°	1
*7*.	The mesial arrowhead should engage the mesio-buccal undercut	1
*8*.	The distal arrowhead should engage the disto-buccal undercut	1
*9*.	The mesial arm of the clasp should follow the occlusal embrasure	1
*10*.	The distal arm of the clasp should follow the occlusal embrasure	1
*11*.	The mesial arm of the clasp should touch the occlusal embrasure	1
*12*.	The distal arm of the clasp should touch the occlusal embrasure	1
*13*.	When the mesial arm goes on the palatal tissue, there should be about 0.5 mm to 1 mm clearance	1
*14*.	When the distal arm goes on the palatal tissue, there should be about 0.5 to 1 mm clearance	1
*15*.	The mesial arm should be bend towards palate in a mesial direction	1
*16*.	The distal arm should be bend towards palate in a mesial direction	1
*17*.	The mesial tag should be facing towards the palate	1
*18*.	The distal tag should be facing towards the palate	1

After submitting the Adams clasp, students were then requested to answer an online students’ satisfaction questionnaire that was newly developed for this study. The questionnaire consisted of a set of demographic questions followed by twenty-nine items grouped under five sections: (1) Infrastructure and materials provided; (2) Video/live demonstration; (3) Teaching method; (4) Wire-bending task; and (5) Efficiency of lecturer and technician during the classroom activity. Responses were recorded using a 5-point Likert scale from strongly disagree to strongly agree with an open-ended question for general feedback after each section. Content and face validation were conducted by a panel of experts which included two orthodontists and an expert in the educational field to determine the suitability of each item. Prior to use, the questionnaire was pre-tested and modified based on feedback by nine third year undergraduate dental students, who were not involved in this study.

The validated questionnaire was completed on an online platform anonymously by the students after each teaching session within one week. Both groups received the same questionnaire with minor substitutions to keywords to reflect the teaching method i.e. ‘live demonstration’ with ‘video demonstration’ and ‘flipped classroom’ with ‘conventional classroom’.

A crossover study was then conducted three weeks later where group LD attended a flipped classroom while group FC attended a live demonstration, on the fabrication of Adams clasp. At this time point, only students’ satisfaction questionnaires were again collected from each student for blinded analysis. Students were not required to submit another wire-bending for assessment at this time point as the students have learnt the skills which are not able to be washed out.

Statistical analysis was conducted using the Statistical Package for Social Sciences (SPSS) Version 25.0. Inter-examiner and intra-examiner reliability were tested using Intraclass Correlation Coefficient (ICC) by using the summed score of the rubric. Mean differences between the two groups in the scores for Adams clasp were assessed using independent samples t-test. During data analysis, the 5-point Likert scale used to measure students’ satisfaction was reclassified to three categories; “Strongly disagree/ Disagree”, “Uncertain”, and “Agree/ Strongly Agree” as the data was not normally distributed. Chi-square and Fisher’s exact test was performed to examine the relation between perceived satisfaction and teaching method. An alpha of 0.05 was used as the level of significance.

## Results

A high degree of reliability was found in the two examiners’ measurements of the quality of Adams clasps. The average measure for inter-examiner ICC was 0.959 with a 95% confidence interval from 0.833 to 0.990 (F (9, 9) = 24.101, p<0.001). The level of reliability for the two individual assessors based on the reported 95% confidence interval of the estimate ICC were also considered excellent at average measures of 0.989 (Examiner 1) and 0.985 (Examiner 2).

The demographics of the subjects are shown in Tables 2 and 3 shows the mean scores for the quality of the Adams clasps between both groups. Forty third year dental students were involved in the study with 85% being female and age ranged between twenty and twenty-two, with a mean age of twenty-one years old. No significant differences were found between group LD and group FC for hand dominance and students’ perceived artistic ability (p value = 0.081 and 0.891 respectively).

**Table 2:** **Sample characteristics**.

		Distributions	
	Total (N = 40)	Group LD (n = 20)	Group FC (n = 20)
	N (%)	n (%)	n (%)
Gender			
Male	6 (15)	3(15)	3(15)
Female	34 (85)	17(85)	17(85)
Ethnicity			
Malay	31(77.5)	15(75)	16(80)
Chinese	7(17.5)	4(20)	3(15)
Other	2 (5)	1(5)	1(5)
Dominant hand			
Right	35 (87.5)	18 (90)	17 (85)
Left	3 (7.5)	0 (0)	3 (15)
Both	2 (5)	2 (10)	0 (0)
Perceived artistic ability			
Good	2 (5)	1 (5)	1 (5)
Moderate	33 (82.5)	17 (85)	16 (80)
Poor	5 (12.5)	2 (10)	3 (15)
	Mean (SD)		
Age (in years)	21.05 (0.316)	21.0 (0.324)	21.1 (0.308)

**Table 3:** Mean score obtained by students of both groups in the blind assessment of the Adams clasp wire-bending exercise.

Group	N	Mean score	Standard Deviation	Standard Error of Mean	*P*-value
LD	20	9.775	3.420	0.765	0.374
FC	20	9.125	3.811	0.852	

*P*-value<0.05 indicates statistical significance.

Response rate for the questionnaire was 100%. Table 4 shows the perceived satisfaction of the teaching methods. Only one statement, “I found the classroom arrangements conducive for the wire-bending activity” was found to be statistically significant with FC teaching method having a higher satisfaction score than LD teaching method (92.5% vs 67.5% respectively, p = 0.010). No significant differences were found between the two teaching methods for other statements (p > 0.05). No sequence effect from the crossover design noted.

**Table 4:** Students’ perception of the live demonstration and flipped classroom methods.

Questions	Teaching Method	N	Disagree/ Strongly Disagree (%)	Uncertain (%)	Agree/ Strongly Agree (%)	Chi-square Test^§^/ Fisher’s Exact Test
**Feedback on infrastructure and materials provided**						
I found the classroom arrangements (positioning of the chairs for group activity, audio-visual facilities etc.) conducive for the wire bending activity	LD	40	22.5	10.0	67.5	.010*
	FC	40	2.5	5.0	92.5	
I found the reading material provided (handout) sufficient for the wire bending activity	LD	40	10.0	32.5	57.5	.365
	FC	40	7.5	20.0	72.5	
I used the reading material provided (handout) for the wire bending activity	LD	40	10.0	35.0	55.0	.282
	FC	40	7.5	20.0	72.5	
I utilised the gold standard Adams clasp placed in the orthodontic lab as reference	LD	40	17.5	35.0	47.5	.796^§^
	FC	40	20.0	40.0	40.0	
**Feedback on the demo video/live demo**						
I found the wire bending steps in the video/live demo clear	LD	40	7.5	5.0	87.5	.204
	FC	40	2.5	17.5	80.0	
I found the wire bending steps in the video/live demo easy to understand	LD	40	7.5	7.5	85.0	.304
	FC	40	2.5	17.5	80.0	
I found the video/live demo a suitable method to learn wire bending	LD	40	7.5	10.0	82.5	.613
	FC	40	7.5	17.5	75.0	
I found the video/live demo useful for the wire bending activity	LD	40	5.0	7.5	87.5	.606
	FC	40	2.5	15.0	82.5	
**Feedback on the teaching method (flipped classroom/conventional classroom)**						
I found the teaching method improved my knowledge of wire bending	LD	40	2.5	17.5	80.0	.790
	FC	40	2.5	25.0	72.5	
I found the teaching method improved my skills of wire bending	LD	40	5.0	20.0	75.0	1.000
	FC	40	2.5	20.0	77.5	
I practiced wire bending outside the classroom	LD	40	7.5	15.0	77.5	.565
	FC	40	2.5	12.5	85.0	
I found the teaching method enjoyable	LD	40	10.0	22.5	67.5	.331
	FC	40	2.5	17.5	80.0	
**Feedback on the task given (fabrication of one Adams clasp)**						
I felt I had acquired sufficient knowledge to fabricate the Adams clasp	LD	40	15.0	22.5	62.5	.106
	FC	40	2.5	17.5	80.0	
I found it easy to fabricate the Adams clasp	LD	40	22.5	42.5	35.0	.377^§^
	FC	40	15.0	35.0	50.0	
I was confident with my performance during the wire bending activity	LD	40	20.0	45.0	35.0	.450^§^
	FC	40	10.0	52.5	37.5	
I was satisfied with my final product (Adams clasp)	LD	40	12.5	52.5	35.0	.122^§^
	FC	40	20.0	30.0	50.0	
I had adequate time to complete my wire bending task	LD	40	2.5	15.0	82.5	.309
	FC	40	7.5	25.0	67.5	
**Feedback on the efficiency of lecturer and technician during the classroom activity**						
I found the lecturer was available for me during the wire bending activity in the classroom	LD	40	7.5	7.5	85.0	.393
	FC	40	0.0	7.5	92.5	
I found the lecturer was helpful for me during the wire bending activity in the classroom	LD	40	5.0	22.5	72.5	.099
	FC	40	0.0	10.0	90.0	
I found the technician was available for me during the wire bending activity in the classroom	LD	40	7.5	0.0	92.5	.241
	FC	40	0.0	2.5	97.5	
I found the technician was helpful for me during the wire bending activity in the classroom	LD	40	5.0	5.0	90.0	.423
	FC	40	0.0	2.5	97.5	

## Discussion

This was a comparative prospective study to assess the effectiveness of the flipped classroom method in comparison to conventional live demonstration in teaching skills of fabricating orthodontic Adams clasp to undergraduate dental students. Flipped classroom has been one of the focal points which have received considerable attention in medical and dental education in recent years [14, 15]. A flipped classroom model for teaching pre-clinical dental skills has been tested and received positively by students [4, 15].

In terms of the skills gained to fabricate Adams clasp, the mean scores of both groups showed no significant difference, thus both groups performed at a similar level of skills. This finding indicates that both teaching methods were equally effective to train students in producing similar quality of Adam clasps. The finding of the current study contradicted many previous studies that showed the superiority of flipped classroom method over conventional classroom method. Several factors may have contributed to this result. Both groups were given a week to work on the fabrication of Adams clasps to reduce bias, the LD group learnt the process and then submitted a week later. Even though the FC group were given access to the online learning a week before the submission date, they may not necessarily have watched the video demonstration as soon as access was given, especially since they are not accustomed to such teaching method. Thus, they may not have optimize the full potential of the flipped classroom method. Secondly, as flipped classroom setting is new to the teaching staff and students, the full potential of it might not have been reached. Improved experience with flipped classroom setting might lead to a more promising result [16]. Nevertheless, our findings agree with a recent systematic review that showed the flipped classroom was as effective as the traditional teaching method [14].

Seating arrangement is important as research has shown that poor seating arrangement can affect the students’ learning [17]. It was hypothesized that students who are seated in proximal seat in their class near to the technicians (teachers) would benefit more and learn better from the wire-bending skill training compared to those located in further seats. Classroom physical setting such as arrangement of the seats and learners seating position in class are believed to have important roles in optimizing the class management [18, 19]. Many studies support the fact that learning is maximized when students are seated near the teacher/instructor even though this was not all the time statistically significant [20–25]. Therefore, it can be concluded that students learn better when they are seated nearer to their teachers, even though not many research supports that. Wannarka & Ruhl (2008) highlighted that classroom seat position may have an impact on the management of the class [19]. In a study done by Halstead (1974), it was concluded that “A student in the classroom is properly seated if he has a clear view of the instructor……” [26]. It must be noted that the seating position can assist or hinder the students’ learning. However, the classroom seat arrangement will be adapted based on students’ needs and nature of the course material to be presented. Suitable seat arrangement will create the chance for the learners to be engaged in their own learning and cooperate with their own course mates.

Traditionally, the classroom setting is limited to three seating arrangements i.e. rows, horseshoe, or clusters due to space limitations [27]. In this study, a cluster seating approach was used for both groups with a ratio of 10 students per technician and per lecturer. In the conventional method, students tended to cluster around the technicians (teachers) to obtain a good view of the wire-bending procedure. Visibility can be poor as the view may be blocked by the angle and the technicians’ hands. This was supported with only about two-third (67.5%) of the students agreeing that the classroom arrangement in LD was conducive for their learning. Whereas, the majority of the students (92.5%) agreed that the classroom arrangement in FC was conducive. The flipped classroom method had allowed the students to modify their personal learning space when they viewed the video demonstration online at their own preferred location, time, and pace. Those who experienced the flipped classroom method were also able to appreciate the physical classroom arrangements for the group activity and audio-visual facilities as compared to conventional method where most of the class time involved watching the live demonstration. This may be because they were able to access the video demonstration using their smartphones and laptops at any seating positions that they were comfortable with, thus being able to engage in their own learning process. These findings are in line with Alqahtani et al. (2015) and Aragon & Zibrowski (2008), in which students in the clinic, who were given a copy of a video, reported that they can see better on video versus a demonstration with everyone crowding around, and that the procedure can be reviewed at any time [2, 28]. In 2013, it was reported that roughly 80% of Malaysian students owned a smartphone or had access to mobile devices and were using these devices to support their learning [29]. It is only natural to start incorporating these devices in the learning environment.

In terms of learning, flipped environment will support more customizable and accessible learning. Lecture material can be accessed to fit the learners’ schedule who is mobile between the clinic and classroom to fulfil the expectation of the course [30]. The focal point in flipped classroom is to create a more student-centred environment, in which students are encouraged to enhance their higher level of learning as advocated in Bloom’s taxonomy [31]. On the other hand, flipped classroom allows lecturers to expose students to problem solving and critical thinking within their classroom allocated time. On the contrary, in conventional teaching, Bloom cognitive levels such as acquiring the knowledge and understanding of the subject happens in the classroom through lectures. The other levels such as application, analysis and evaluation will take place outside of the classroom environment with little guidance from the teachers. In terms of space, students have the chance to watch the lecture at their own pace, they can revise the concepts that they did not understand repeatedly until they can master it, and then they can discuss the complicated concept with their peers in class to ensure they develop the skills that they did not manage to grasp.

Though not asked in this study, the impact of the classroom arrangements between the two groups may subsequently raise the issue if attendance should be compulsory for those who were given the video demonstration. When Alqahtani et al. (2015) compared live demonstration and practical video, they found no differences in the student’s preference for learning as well as the performance in the wire bending of Adam’s clasps. However, in that study, the students would not benefit from the flipped classroom concept where students would get opportunities for face-to-face teaching after the practical video [2]. Nonetheless, in another study comparing flipped and conventional teaching, 68.7% medical students felt that in-class practice activities for flipped sessions should be non-mandatory [32].

## Limitations

FC method requires students’ initiative for active learning. The study provided standardised instructions and the online learning materials for the students to learn prior to the in-class classroom session. However, the benefit of FC session will be limited should the students not follow the instructions or did not utilise the video demonstration online fully. Future studies may assess students’ utilisation of the online learning materials provided, including the pause, rewind, and fast-forward function, to investigate its association with the effectiveness of FC approach. Hand dominance and students’ perceived skill levels in arts and crafts may have been confounding factors of the study but baseline data analysis revealed no significant differences between group LD and group FC for these variables.

## Conclusions

Students perceived the classroom arrangements in the flipped classroom method as significantly more conducive for the wire-bending teaching activity. Within the limitations of the studies, flipped classroom method is equally as effective as a conventional live demonstration method in transferring orthodontic wire-bending skills for fabrication of Adams clasp.

## References

[1] Packer ME, Scott BJ, Davis DM. An assessment of the influence of clinical demonstrations on the confidence of undergraduate dental students, when treating patients requiring removable partial dentures. Eur J Dent Educ. 1999;3: 133–9. doi: 10.1111/j.1600-0579.1999.tb00079.x 10865348

[2] Alqahtani ND, Al-Jewair T, Khalid AM, Albarakati SF, ALkofide EA. Live demonstration versus procedural video: a comparison of two methods for teaching an orthodontic laboratory procedure. BMC Medical Education. 2015;15: 199–203. doi: 10.1186/s12909-015-0479-y 26537393 PMC4634912

[3] Packer ME, Rogers JO, Coward TJ, Newman PS, Wakeley R. A comparison between videotaped and live demonstrations, for the teaching of removable partial denture procedures. Eur J Dent Educ. 2001;5: 17–22. doi: 10.1034/j.1600-0579.2001.005001017.x 11168489

[4] Park SE, Howell TH. Implementation of a flipped classroom educational model in a predoctoral dental course. Journal of Dental Education. 2015;79: 563–70. 25941150

[5] Bishop JL, Verleger MA. The flipped classroom: A survey of the research. In ASEE national conference proceedings, Atlanta, Georgia 2013 (Vol. 30, No. 9, pp. 1–18).

[6] Hawks SJ. The flipped classroom: now or never? Aana J. 2014;82: 264–9. 25167605

[7] Tucker B. The flipped classroom. Educ Next. 2012;12: 82–3.

[8] Chung CJ, Lai CL, Hwang GJ. Roles and research trends of flipped classrooms in nursing education: a review of academic publications from 2010 to 2017. Interactive Learning Environments. 2019;22: 1–22.

[9] Lin HC, Hwang GJ, Hsu YD. Effects of ASQ-based flipped learning on nurse practitioner learners’ nursing skills, learning achievement and learning perceptions. Computers & Education. 2019;139: 207–21.

[10] Lin HC, Hwang GJ. Research trends of flipped classroom studies for medical courses: A review of journal publications from 2008 to 2017 based on the technology-enhanced learning model. Interactive Learning Environments. 2019;27(8): 1011–27.

[11] Gianoni‐Capenakas S, Lagravere M, Pacheco‐Pereira C, Yacyshyn J. Effectiveness and perceptions of flipped learning model in dental education: a systematic review. Journal of dental education. 2019;83(8): 935–45. doi: 10.21815/JDE.019.109 31133621

[12] Strelan P, Osborn A, Palmer E. The flipped classroom: A meta-analysis of effects on student performance across disciplines and education levels. Educational Research Review. 2020;30:100314.

[13] Vanka A, Vanka S, Wali O. Flipped classroom in dental education: A scoping review. European journal of dental education: official journal of the Association for Dental Education in Europe. 2019. doi: 10.1111/eje.12487 31808231

[14] Chen F, Lui AM, Martinelli SM. A systematic review of the effectiveness of flipped classrooms in medical education. Med Educ. 2017;51: 585–97. doi: 10.1111/medu.13272 28488303

[15] Crothers AJ, Bagg J, McKerlie R. The Flipped Classroom for pre-clinical dental skills teaching–a reflective commentary. British Dental Journal. 2017;222: 709. doi: 10.1038/sj.bdj.2017.409 28496219

[16] Gordy XZ, Zhang L, Sullivan AL, Haynie L, Richards-Moore L, Bailey JH. A multi-disciplinary empirical investigation of active learning classroom’s effects on student learning. Interdisc Educ Psychol. 2018;2: 1–8.

[17] Black S. Achievement by design. American School Board Journal. 2007;194: 39–41.

[18] MacAulay DJ. Classroom environment: A literature review. Educational Psychology. 1990;10: 239–53.

[19] Wannarka R, Ruhl K. Seating arrangements that promote positive academic and behavioural outcomes: A review of empirical research. Support for Learning. 2008;23: 89–93.

[20] Schwebel AI, Cherlin DL. Physical and social distancing in teacher-pupil relationships. Journal of Educational Psychology. 1972;63: 543.

[21] Wulf KM. Relationship of assigned classroom seating area to achievement variables. Annual meeting of the American educational research association, San Francisco 1976 (pp. 2–13).

[22] Stires L. Classroom seating location, student grades, and attitudes: Environment or self-selection?. Environment and Behavior. 1980;12: 241–54.

[23] Perkins KK, Wieman CE. The surprising impact of seat location on student performance. The Physics Teacher. 2005;43: 30–3.

[24] Bailenson JN, Yee N, Blascovich J, Beall AC, Lundblad N, Jin M. The use of immersive virtual reality in the learning sciences: Digital transformations of teachers, students, and social context. The Journal of the Learning Sciences. 2008;17: 102–41.

[25] Meeks MD, Knotts TL, James KD, Williams F, Vassar JA, Wren AO. The impact of seating location and seating type on student performance. Education Sciences. 2013;3: 375–86.

[26] Halstead DK. Statewide planning in higher education. Washington, D.C.: U.S. Government Printing Office 1974 (pp. 485,505–507).

[27] Weinstein CS. Designing the instructional environment: Focus on seating. Bloomington, IN: Proceedings of selected research and development presentations at the convention of the Association for Educational Communications and Technology 1992 (ERIC Document Reproduction Service No. ED 348 039).

[28] Aragon CE, Zibrowski EM. Does exposure to a procedural video enhance preclinical dental student performance in fixed prosthodontics?. Journal of Dental Education. 2008;72: 67–71. 18172237

[29] Song HS, Murphy A, Farley H. Mobile devices for learning in Malaysia: Then and now. In ASCILITE-Australian Society for Computers in Learning in Tertiary Education Annual Conference 2013 (pp. 830–834). Australasian Society for Computers in Learning in Tertiary Education.

[30] Williams DE. The future of medical education: flipping the classroom and education technology. The Ochsner Journal. 2016;16: 14–5. 27026786 PMC4795492

[31] Krathwohl DR. A revision of Bloom’s Taxonomy: An overview. Theory into Practice. 2002;41: 212–8.

[32] Pettit RK, McCoy L, Kinney M. What millennial medical students say about flipped learning. Adv Med Educ Pract. 2017;8: 487–97. doi: 10.2147/AMEP.S139569 28769600 PMC5529113

